# Identification of Displaced Endometrial Glands and Embryonic Duct Remnants in Female Fetal Reproductive Tract: Possible Pathogenetic Role in Endometriotic and Pelvic Neoplastic Processes

**DOI:** 10.3389/fphys.2012.00444

**Published:** 2012-12-03

**Authors:** Jean Bouquet de Jolinière, Jean Marc Ayoubi, Guy Lesec, Pierre Validire, Alexandre Goguin, Luca Gianaroli, Jean Bernard Dubuisson, Anis Feki, Jean Gogusev

**Affiliations:** ^1^Department of Gynecology, Fribourg HospitalFribourg, Switzerland; ^2^Department of Gynecology, Foch HospitalSuresnes, France; ^3^SIPATH-Institut de Cytologie et d’Anatomie PathologiquesClermont Ferrand, France; ^4^Department of Pathology, Institute Mutualiste MontsourisParis, France; ^5^INSERM U567 Hôpital NeckerParis, France; ^6^Reproductive Medicine Unit, Società Italiana di Studi di Medicina della RiproduzioneBologna, Italy

**Keywords:** fetus, endometriosis, neoplastic process, ectopic glands, immunohistochemistry

## Abstract

**Background:** Recent findings strongly promoted the hypothesis that common pelvic gynecological diseases including endometriosis and ovarian neoplasia may develop *de novo* from ectopic endometrial-like glands and/or embryonic epithelial remnants. To verify the frequency, the anatomical localization and the phenotype of misplaced endometrial tissue along the fetal female reproductive tract, histological and immunohistochemical analyses of uteri, fallopian tubes, and uterosacral ligaments were performed. **Methods:** Reproductive organs were collected from seven female fetuses at autopsy, five of them from gestational ages between 18 and 26 weeks and two fetuses with gestational ages of 33 and 36 weeks deceased of placental anomalies. Serial sections from areas containing ectopic glands and embryonic duct residues were analyzed by histological and immunohistochemical procedures. **Results:** Numerous ectopic endometrial glands and stroma were detected in the myometrium in two fetuses with low levels of expression of estrogen receptor-alpha (ER-α) and progesterone receptors (PR). The embryonic ducts were localized in the uterine broad and ovarian ligaments and under the fallopian tube serosa in six fetuses. Low levels of steroid receptors expression were found in the embryonic residues, whereas the carcino-embryonic antigen (CEA) and the tumor marker Ca 125 were not detected. The embryonic residues stromal component strongly expressed the CD 10 and vimentin proteins. **Conclusion:** The anatomical and the immunohistochemical features of the ectopic organoid structures identified in fetal female reproductive tract suggest that endometriotic as well as neoplastic disease in adult women may develop on the basis of misplaced endometrial glands and/or embryonic cell remnants.

## Introduction

Endometriosis is a heterogeneous gynecological disease clinically characterized by the presence of different anatomo-clinical subtypes (Giudice, 2010). The most frequently proposed pathogenetic mechanism is tubal regurgitation during menstrual cycle, which however cannot explain all clinical forms of this disease (Sampson, 1927; Bulun, 2009). Indeed, occurrence of endometriosis was described in patients with Rokitansky–Kuster–Hauser syndrome who does not have functioning endometrial tissue (Acien, 1986; Cho et al., 2009) as well as in male patients with endometriosis of the prostate, bladder, and the abdominal wall (Schrodt et al., 1980; Beckman et al., 1985; Martin and Hauck, 1985). In this regard, the theory of transformation of the vestigial tissue of Müllerian or Wolfian origin and the coelomic metaplasia theory can explain the origin of distinct entities of endometriotic lesions as well as development of particular types of ovarian neoplasms (Ridley, 1968; Suginami, 1991; Varma et al., 2004; Mandai et al., 2009; Wei et al., 2011). In the same context, a recent study has proposed the fetal origin of endometriosis, that could develop on the basis of altered migration of primitive endometrial tissue during embryogenesis (Signorile and Baldi, 2010). These authors assessed that the incidence of the dislocated embryonic structures in fetuses is similar to that of endometriosis occurring in the adult female population (Signorile and Baldi, 2010). In the same direction, relationship between endometriosis and malignancies arising in gonadal and extragonadal endometrial implants become supported by several clinical pathologic and molecular investigations (Brinton et al., 1997; Vercellini et al., 2000; Varma et al., 2004; Prowse et al., 2006; Wei et al., 2011). These studies suggested that histogenetically, endometriosis represents an important site of origin of ovarian and other pelvic malignancies (Vercellini et al., 2000; Mandai et al., 2009; Wei et al., 2011). It was described that such neoplasms are constituted of clear epithelial cells and tend to be detected in earlier stages, their prognosis being more favorable (McMeekin et al., 1995; Wei et al., 2011). In addition, embryonic duct remnants were often microscopically observed adjacent to ovarian tumors as well as close to pelvic endometriotic lesions suggesting a pathogenetic relationship (Rudgers and Scully, 1988; Mai et al., 1998; Dubeau, 2008; Nissenblatt, 2011).

In the present study, we evaluated the incidence and the anatomical location of displaced endometrial tissue in the reproductive tract in seven female fetuses. Serial sectioning of the reproductive organs was realized followed by immunohistochemical analysis of tissue areas containing ectopic glands and embryonic cell rests. It was observed that the anatomical and the phenotypic features of the mislocated tissue components recall some pathological characteristics of both benign and malignant gynecological conditions.

## Material and Methods

### Tissue preparation

Reproductive organs from a series of seven human female fetuses at different gestational ages ranging from 18 to 36 weeks were obtained at autopsy. All together, the causes of fetal death were placental pathology in two samples, cardiac malformations in two cases and voluntary abortions in three. The reproductive organs were carefully dissected, fixed in buffered formaldehyde, and included in paraffin.

Between 200 and 400 serial sections with thickness of 5–7 mm from each paraffin block containing uteri, fallopian tubes, ovaries, and uterosacral ligaments were prepared and stained by hematoxylin and eosin (H&E). To ascertain tissue sections containing ectopic endometrial glands and/or embryonic duct remnants, every sixth slide was separately stained and microscopically analyzed.

### Antibodies

The following antibodies were employed; rabbit anti estrogen receptor-alpha (ER-α), (cat N° sc-54, Santa Cruz Biotechnology Inc., Santa Cruz, CA, USA); monoclonal mouse anti human progesterone receptor, clone PR 636 (Dako Laboratories Glostrup, Denmark); monoclonal anti human CA125, clone M11 CA125 (Dako); monoclonal anti human CD10 clone 56C6 (Dako); monoclonal anti human carcino-embryonic antigen (CEA) clone II-7 (Dako); rabbit anti human alpha-1-foetoprotein (Dako); monoclonal anti human epithelial membrane antigen (EMA) clone E29 (Dako); mouse anti human Cytokeratin 7 (clone RCK 105, cat N° sc-23876, Santa Cruz Biotechnology Inc., Santa Cruz, CA, USA) and monoclonal mouse anti-vimentin clone VIM 3B (Dako).

### Immunohistochemistry

Five micrometres thick sections were deparaffinized in xylene, rehydrated through graded alcohol series, and washed in PBS. For antigen retrieval, the slides were immerged in citrate buffer (pH = 6.0) during 25 min at 96°C essentially as described (Hsu et al., 1981). After brief wash in PBS, the appropriate dilution of the primary antibodies was applied on slides during 1 h. Immunoreactivity was revealed using the avidin-biotin complex method (LSAB2 System HRP, Dako, Denmark) with 3,3′-diaminobenzidine tetra hydrochloride (DAB) as a chromogen. After checking the staining intensity, the sections were washed in water and counterstained with Harris hematoxylin (Sigma-Aldrich Chimie Sarl, Saint-Quentin Fallavier, France). The slides were then dehydrated in ascending grades of ethanol and after clearing the sections with xylene mount and they were covered with DPX mountant (Merck Chimie SAS, Fontenay sous Bois, France). Negative controls consisted of replacement of the primary antibodies with non-immune mouse or rabbit serum or buffer alone. The extent and the intensity of the staining were determined by the objective observer procedure. Epithelial staining intensity was graded on a 0–3 scale, where 0, no staining was assessed with anti-rabbit secondary antibody alone; 1, weak; 2, moderate; and 3, intense staining. The percentage of immunoreactive cells was obtained by counting the number of stained cells from a total of 200 cells at magnification of ×20, composing the ectopic endometrial glands and stroma or embryonic duct structures and stroma of each case and from each location.

## Results

**Figure 1:**
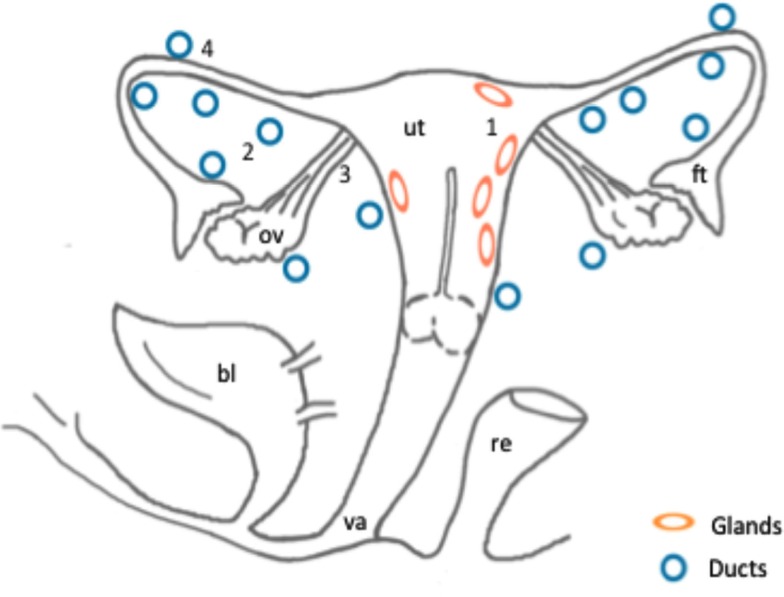
**Schematic representation of the anatomical localization of ectopic endometrial glands and embryonic duct remnants along the reproductive tract of six female fetuses**. Areas of the distribution of ectopic glands in the myometrium (1) in two fetuses: (ellipses). Distribution of embryonic ducts in the uterine broad ligaments (2), ovarian ligaments (3), and under the fallopian tube serosa (4) in a total of six fetuses: (circles). Abbreviations: ut, uterus; bl, bladder; va, vagina; ov, ovary; ft, fallopian tube; re, rectum.

**Figure 2:**
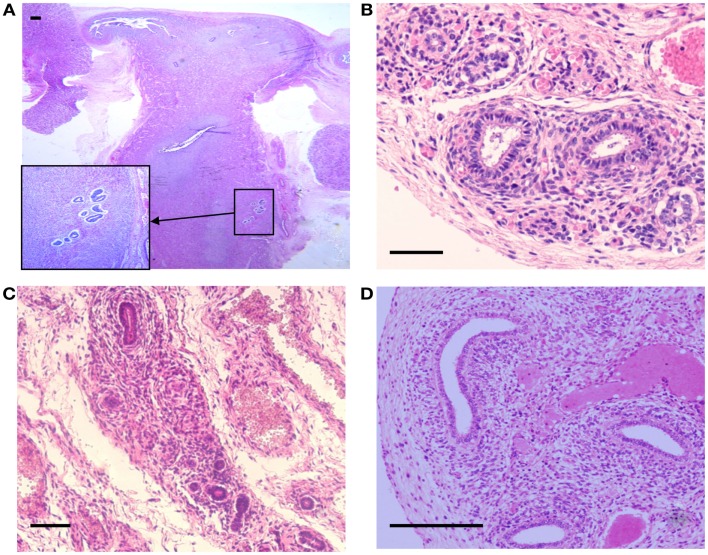
**Hematoxylin and Eosin stained sections with areas of ectopic endometrial glands and embryonic ducts**. Histological appearance of ectopic glands and stroma observed (insert at higher magnification) in fetal uterine wall **(A)**. Presence of embryonic ducts located in the broad ligament **(B)**, under the fallopian tube serosa **(C)**, and ducts located in the ovarian ligament **(D)**. Note presence of a stromal component surrounding the duct residues in **(A–D)**. Scale bars, 100 μm.

Tissue sections of the reproductive organs from all fetuses stained by hematoxylin an eosin showed normal anatomical morphology and histological structure with asymmetrical uterine endometrial branching invaginations, while inflammatory or fibrotic areas or hemorrhage were not detected. In the fetuses of the lower gestational age, the uterine central cavities appeared lined by columnar epithelial cells and were devoid of differentiated glandular structures. The uterine cavity of the 33 and 36 weeks old fetuses was lined by epithelial cells forming rare immature glandular structures of different sizes. Remarkably, ectopic foci of glandular structures surrounded by a densely distributed stromal cells were found in serial sections of the uterine myometrium in two fetuses, with gestational ages of 25 weeks and of 36 weeks (Figure 1). The histological structure of the ectopic glands predominantly showed a single layer of columnar cells similar to the endometrial epithelial lining with basal nuclei and mucin containing cytoplasm. Some of the misplaced glands of larger size showed focal cytological atypia but mitotic figures were not observed. Histological appearance of the ectopic glands distributed in the uterine wall of the 36-week-old fetus is shown in Figure 2A. Of note, distinct large foci of epithelial cellular elements forming tubular gland like structures were present in the ovarian hilus in one fetus with gestational age of 23 weeks (not shown).

The second form of ectopic structures observed were the embryonic tubular duct formations located bilaterally in the broad uterine and ovarian ligaments in five tissue samples and under the fallopian tube serosa in one fetus, some of them being surrounded by a dense endometrial-like stroma (Figure 1; Figures 2B–D). In general, the embryonic structures present in the uterine and ovarian ligaments histologically appeared as discontinuous segments of tortuous ducts surrounded by a rich vascular network. Each duct remnant exhibited a lumen lined by cuboidal cells, surrounded by a layer of mesenchymal stroma like component. Interestingly, in the 36 weeks old fetus, both embryonic ducts located in the broad ligament and ectopic glands embedded in the myometrium were simultaneously observed.

**Figure 3:**
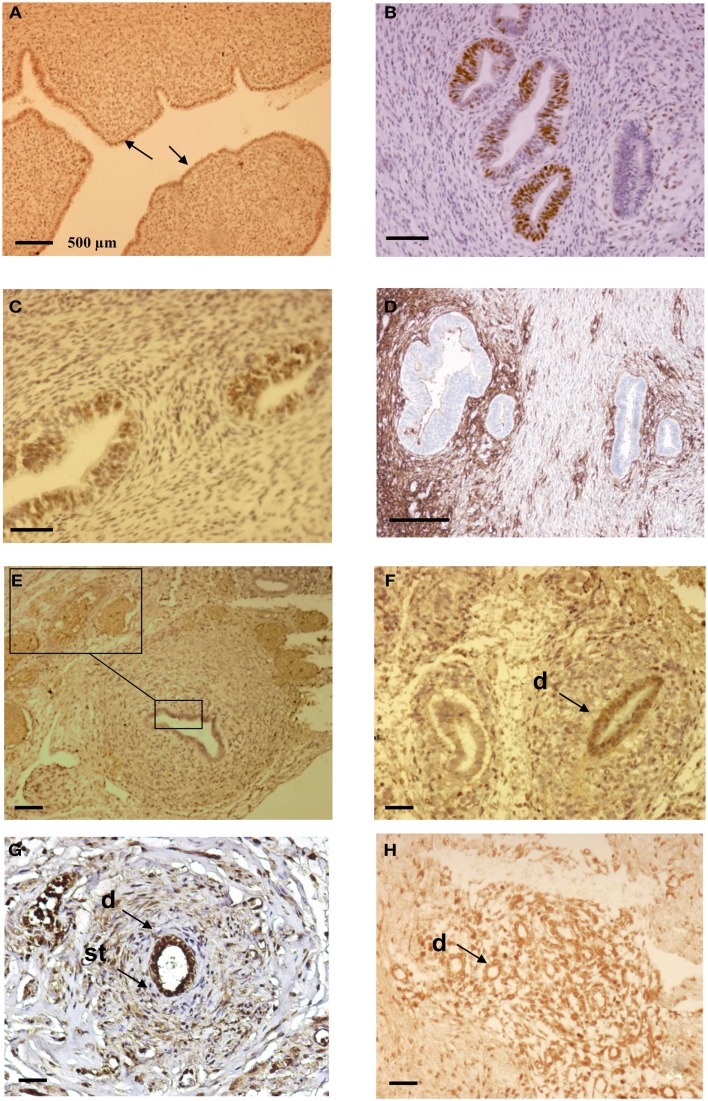
**Immunohistochemical analysis of ectopic endometrial glands and embryonic ducts in fetal reproductive tract**. Immunolabeling with anti PR antibody of uterine cavity wall lining cells [**(A)**, arrows]. Immunostaining of the fetal ectopic glands located in the uterine myometrium with anti PR **(B)**, anti ER-α **(C)**, and anti CD10 antibodies **(D)**. Expression of the PR in embryonic duct located in the ovarian ligament; insert at higher magnification **(E)**, expression of ER-α in a duct located under the fallopian tube serosa **(F)**, and expression of alpha-1-fetoprotein **(G)** and CD10 molecules **(H)**. D = duct; St = stromal layer. Scale bars; 500 μm in **(A)** and 100 μm in **(B–H)**.

By immunohistochemistry, various levels of several marker antigens were detected in both the ectopic uterine glands and embryonic ducts. As a rule, a higher level of the EMA, PR, and ER-α molecules were detected in cells lining the uterine cavity with a mean of 64, 53, and 21% of labeled elements respectively. A lower percentage of immunoreactivity for EMA (23%), PR (14%), and ER-α (6%) was revealed in the ectopic glandular structures localized in the uterine wall. A strong expression of CD10 (41.3%) but lower level of vimentin specific immunolabeling (20%) was observed in the stromal component surrounding the displaced glands. Representative illustrations of PR expression in orthotopic endometrial cells as well as the expression of PR, ER-α, and CD10 in the uterine ectopic glands and stroma are shown in Figures 3A–D.

Concerning the embryonic remnants, consistent EMA immunostaining ranging between 32 and 53% was detected in the duct lining cells, while lower level of expression in duct cells of both ER-α (10–23%) and PR (13–44%) molecules was found in all samples. The stromal-like component surrounding the duct residues contained between 1 and 3% of immunoreactive cells for ER-α and between 4 and 13% of immunoreactive cells for PR. Approximately 2–11% of the ductal cells and 1–3% of the stormal cells expressed cytokeratin 7, while CEA and CA125 molecules were not detected. Using anti alpha-1-fetoprotein antibody, close to 42% of the epithelial and duct surrounding mesenchymal cells were intensely immunoreactive on sections from two fetuses. Representative illustrations of the embryonic duct residues labeled with anti ER-α, anti PR, anti alpha-1-fetoprotein and anti CD10 antibodies are shown in Figures 3E–H. The mean values of the percentage of imunoreactive embryonic duct lining cells and stroma against EMA, Cytokeratin 7, ER-α, PR, vimentin and CD10 antigens are reported in Table 1.

**Table 1:** **Summary of the percentages of immunoreactive cells in embryonic ducts and the surrounding stroma present in various locations of the fetal reproductive tract**.

Gestational age (weeks)	EMA*		Cytokeratin 7*		ER*		PR*		Vimentin*		CD10*	
	D	St	D	St	D	St	D	St	D	St	D	St
18	32	−	2	−	10	−	19	11	35	4	2	55
19	52	−	−	−	12	−	21	6	26	9	−	38
20	38	−	−	3	18	3	13	8	34	13	−	42
21	43	−	−	−	13	−	19	4	22	20	−	39
22	46	−	−	−	23	−	31	13	25	6	−	44
32	0	0	0	−	0	0	0	0	0	0	0	0
36	53	1	11	1	16	1	44	9	53	32	4	64
Mean value**	264	1	13	4	92	4	147	51	195	84	6	282

D, embryonic duct; St, Stromal layer; *, percentage of cells expressing the indicated marker; mean value** [represents a ratio of the number of counted cells (200) divided by the number of immunolabeled cells].

## Discussion

In this study we show presence of misplaced endometrial glands and embryonic duct-like remnants in the reproductive organs in six of seven examined female fetuses. The phenotypic features of the ectopic glands in the myometrium of two fetuses indicate particularly weak expression of PR and ER-α steroid hormone receptors in comparison to their high level of expression in cells lining the endometrial cavity. In these two cases, moderate level of PR expression was also revealed in the cell nuclei of the stromal component, while ER-α receptors were not found. Overall, the presented findings are in accordance with other studies describing the levels of ER-α and PR in misplaced glands in patients with both endometriosis and adenomyosis (Van der Walt et al., 1986; Ferenczy, 1998; Bulun et al., 2010). In fact, inconsistent results concerning the pattern of steroid hormones receptor expression in fetal female genital tissues were reported by several studies (Glatstein and Yeh, 1995; Brandenberger et al., 1997). For example, in adult patients, decreased expression of both ER-α and PR were reported in seven cases of ovarian endometriomas in comparison to their consistent levels in the autologous endometrial cells (Tamaya et al., 1979). This is in agreement with other observations indicating that adenomyotic nodules located in the vaginal fornix and the rectovaginal septum do not express PR and ER-α suggesting that they may originate from undifferentiated Müllerian residues (Nisolle and Donnez, 1997; Donnez et al., 2003). Another study reported equal levels of expression of ER and PR receptors in both autologous endometrium and the adenomyotic lesions in adult patients (Ferenczy, 1998).

Concerning the foci of embryonic duct remnants observed, moderate expression of PR and low levels of ER-α were uniformly revealed in the epithelial cells in all cases. Histologically, some ducts appeared dysplastic and surrounded by a dense cellular stromal layer consistently expressing vimentin and CD10 molecules. Of interest, these tubular structures in most of the samples did not clearly express cytokeratin 7, and Ca 125 protein molecules. This might be related to particular phenotypic features of coelomic metaplastic cell rests at given gestational age (Batt and Smith, 1989; Fujii, 1991). Comparatively, consistent level of expression of both estrogen receptor and CA 125 antigens was described in displaced organoid structures in fetal rectovaginal septum, the Douglas pouch, the rectal tube, and at the posterior wall of fetal uteri (Signorile et al., 2009).

In spite of the limited number of studied cases, the findings of ectopic glands and/or embryonic ducts in the reproductive tract of female fetuses is a remarkable phenomenon that could be referred to the theory of involvement of Müllerian or Wolfian cell rests in the pathogenesis of both endometriosis and particular pelvic malignancies (Fujii, 1991; Redwine, 1998, 2003; Leiserowitz et al., 2003; Batt et al., 2007; Signorile et al., 2009; Wei et al., 2011). At present it is considered that a multitude of ovarian, adnexal, and pelvic masses originate from the secondary Müllerian system. On the other hand, it is well known that the coelomic epithelial cells and the accompanying mesenchymal component referred as secondary Müllerian system, have the potential to differentiate toward a Müllerian-directed epithelium and stroma (Fujii, 1991; Mai et al., 1997). Thus, it has been hypothesized that aberrant migration of Müllerian ducts could cause spreading of embryonic cells along the migratory pathway during fetal organogenesis with potential to induce lesions including both endometriosis and ovarian neoplasms (Fujii, 1991; Redwine, 1998; Varma et al., 2004; Batt et al., 2007; Mandai et al., 2009; Signorile et al., 2009). Our data are in relation with another study that described presence of endometriotic foci adjacent to the embryonic duct remnants of coelomic origin involving ovaries and fallopian tubes in three among 18 adult patients (Mai et al., 1998). Phenotypically, the epithelial cells of the duct remnants appeared transformed and showed a diffuse but weak immunoreactivity for estrogen receptor (Mai et al., 1998). The findings of embryonic ducts spread in broad ligaments and under the fallopian tube serosa in our series could be related to studies describing occurrence of endometriomas as well as neoplasms of a Müllerian origin in these locations (Zacharia and O’Neill, 2006; Handa et al., 2007; Wei et al., 2011). Finally, the aberrant and consistent expression of alpha-1-fetoprotein revealed in the embryonic ducts in two fetuses is in accordance with other studies that reported *de novo* expression of this protein in ovarian malignancy arising from endometriosis (Horiuchi et al., 1998; Certin et al., 2007; Takahashi et al., 2011).

In conclusion, the presented data support the theory that at least some subtypes of endometriotic and gynecological neoplastic lesions may be related to anomalies occurring during the embryogenesis. These data stand in relationship with the embryological origin of certain pelvic malignancies based on the metaplastic potentiality of the secondary Müllerian system. The observed frequency of displaced embryonic structures, also suggests a complex pathogenetic mechanism in the development of endometriosis-associated neoplasms including genetic, hormonal, and/or environmental events. Consequently, further studies of endometriotic and neoplastic lesions should include novel embryonic cellular phenotypic markers, that could provide important diagnostic and predictive information to guide clinical decision making.

## Conflict of Interest Statement

The authors declare that the research was conducted in the absence of any commercial or financial relationships that could be construed as a potential conflict of interest.
